# Older Adults’ Economic Security: Do Marital Histories Contribute to Cumulative (Dis)advantages?

**DOI:** 10.1093/geroni/igaf122.559

**Published:** 2025-12-31

**Authors:** Deborah Carr

**Affiliations:** Boston University, Boston, Massachusetts, United States

## Abstract

Social Security benefits rules privilege married persons and penalize divorced, prematurely widowed, and lifelong single older adults. Program rules may be an engine driving cumulative (dis)advantage, because marriage is increasingly an institution of economically privileged persons. Women are more likely than men to be divorced or prematurely widowed, increasing their vulnerability to late-life economic insecurity. We examine Social Security and household income, and poverty rates of older adults based on marital categories aligned with Social Security benefits rules: (re)married; divorced (after short vs. long marriage), widowed (before vs. after age 65), and never married. Data are from the Wisconsin Longitudinal Study, which tracked white high school graduates from ages 18 (1957) to 72 (2011). Our analytic sample includes 5,269 persons (2,498 men and 2,711 women). We used OLS and logistic regression to estimate Social Security income, household income, and poverty status at age 72, adjusted for covariates. Fully adjusted models show that married older adults have higher Social Security and household income and lower poverty rates than all unmarried categories. Divorced women, regardless of marital duration, fare worst on all outcomes. Prematurely widowed persons are worse off than those widowed at older ages. Never married men are less financially secure than all other men. Revisions to Social Security including caregiver credits for years in which a worker had no/low earnings could mitigate disparities in late-life economic security. Our results suggest that cumulative dis/advantage theories should more fully address the role of marital and family roles as engines driving late-life inequalities.

